# Antibody-antigen kinetics constrain intracellular humoral immunity

**DOI:** 10.1038/srep37457

**Published:** 2016-11-24

**Authors:** Maria Bottermann, Heidrun Elisabeth Lode, Ruth E. Watkinson, Stian Foss, Inger Sandlie, Jan Terje Andersen, Leo C. James

**Affiliations:** 1 Medical Research Council Laboratory of Molecular Biology, Division of Protein and Nucleic Acid Chemistry, Francis Crick Avenue, Cambridge, CB2 0QH, United Kingdom; 2 Centre for Immune Regulation, Department of Biosciences, University of Oslo, N-0371 Oslo, Norway; 3 Centre for Immune Regulation, Department of Immunology, Oslo University Hospital Rikshospitalet, University of Oslo, N-0424 Oslo, Norway; 4 Icahn School of Medicine, Mount Sinai, New York, USA

## Abstract

During infection with non-enveloped viruses, antibodies stimulate immunity from inside cells by activating the cytosolic Fc receptor TRIM21. This intracellular humoral response relies on opsonized viral particles reaching the cytosol intact but the antigenic and kinetic constraints involved are unknown. We have solved the structure of a potent TRIM21-dependent neutralizing antibody in complex with human adenovirus 5 hexon and show how these properties influence immune activity. Structure-guided mutagenesis was used to generate antibodies with 20,000-fold variation in affinity, on-rates that differ by ~50-fold and off-rates by >175-fold. Characterization of these variants during infection revealed that TRIM21-dependent neutralization and NFκB activation was largely unaffected by on-rate kinetics. In contrast, TRIM21 antiviral activity was exquisitely dependent upon off-rate, with sub-μM affinity antibodies nevertheless unable to stimulate signaling because of fast dissociation kinetics. These results define the antibody properties required to elicit an efficient intracellular immune response during viral infection.

Intracellular humoral immunity is a recently discovered part of the immune response in which antibodies activate antiviral functions inside the cytosol of infected cells. During this process, antibodies that opsonize viral particles are carried into the cell during infection, where they are detected by the cytosolic Fc receptor TRIM21^1^. TRIM21 is distinct from classical Fcγ receptors in that it is expressed in all tissues and not just hematopoietic cells, highly conserved across mammals and the highest affinity antibody receptor in humans^2^,^3^. An important functional distinction between these different Fc receptors is that TRIM21 actively neutralizes infectious pathogens rather than facilitating the uptake of potentially non-infectious immune complexes for processing by professional antigen presenting cells.

When TRIM21 detects invading virus-antibody complexes in the cytosol, it catalyses the synthesis of multiple ubiquitin chain types to drive a dual sensing and effector response. Ubiquitination by TRIM21 recruits the proteasome and the AAA ATPase VCP, which results in rapid degradation of the viral complex and thereby prevents viral replication within the cell^3^,^4^. Simultaneous with this degradation process, K63 ubiquitin chains released from TRIM21 by the 19S-associated deubiquitinase Poh1 stimulate NFκB, AP-1 and IRF3 immune transcription pathways, leading to potent activation of pro-inflammatory immunity^5^,^6^. TRIM21 antiviral activities have been demonstrated during infection by non-enveloped viruses from diverse families including *adenoviridae, caliciviridae* and *picornaviridae* and the intracellular bacteria Salmonella^6^,^7^,^8^. TRIM21 function provides an important component of protective antibody immunity *in vivo*. Recent data has shown that TRIM21 uses antibodies to elicit a rapid interferon response in a mouse model of infection^9^. Furthermore, animals deficient in TRIM21 were shown to be significantly more susceptible to fatal infection by mouse adenovirus-1 (MAV-1)^10^.

In contrast to the processes that occur after TRIM21 activation, little is known about what factors determine whether antibody-virus immune complexes will trigger a response. The process by which antibody-bound viruses enter the cytosol is entirely dependent upon the virus; it mediates capture on the cell surface, endocytosis and importantly entry to the cytosol. However, the properties that naturally occurring and engineered therapeutic antibodies require in order to accompany the virus and efficiently engage TRIM21 are poorly understood. Here we investigated the antigenic features associated with a potent TRIM21-dependent anti-human adenovirus 5 (Adv5) IgG1 antibody and determined the kinetic requirements for efficient stimulation of antiviral activity upon infection.

## Results

**Figure 1:**
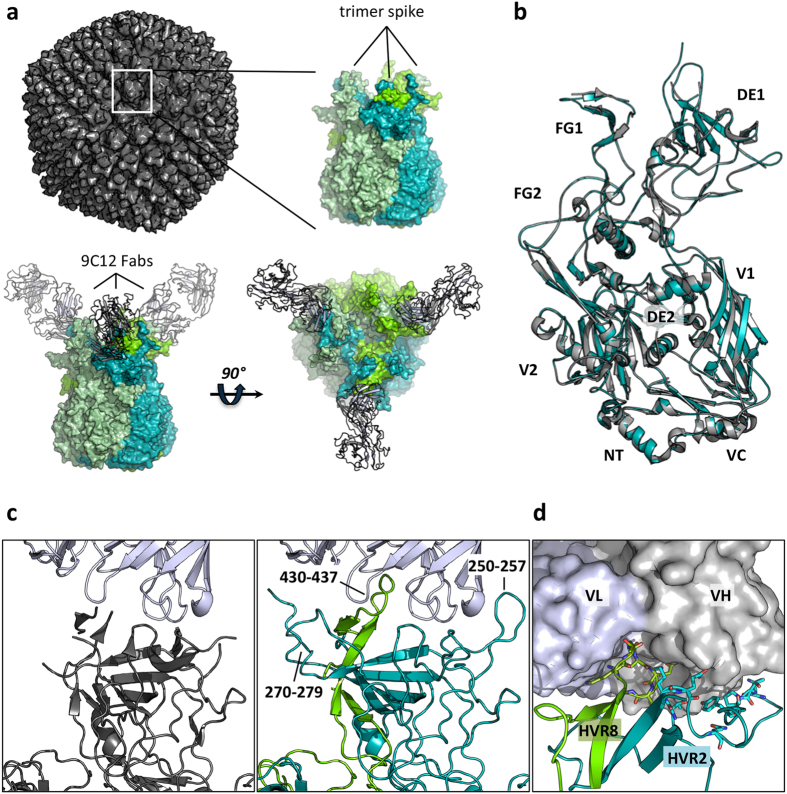
9C12 Fab: Adv5 hexon complexed structure. (**a**) Model of the adenovirus virion based on PDB 1P30 as a gray surface. Surface representations of the hexon trimer from the complexed structure (monomers shown in shades of green). Secondary structures of the bound 9C12 Fabs are in light gray. (**b**) Superposition of free (gray; PDB 1P30) and complexed hexon (green) monomers with labeled domains. The view is from the interior of the molecule, perpendicular to the three-fold molecular axis. (**c**) Side by side images of the interface between 9C12 Fab (gray-blue) and hexon, either showing superposed free structure (gray) or complexed (green). The hexon loops only present in the complexed structure are labeled. Note that two hexon monomers are involved at the interface (light and dark green). (**d**) Surface representation of 9C12 Fab V_L_ (blue) and V_H_ (gray) bound to HVR2 and HVR8 from two different hexon monomers. Residues from HVR loops are shown as sticks.

The anti-Adv5 antibody 9C12 targets hexon, the primary capsid component, which assembles into 240 trimers to form the faces of each icosahedral virion. Previously, we have shown that 9C12 is capable of eliciting a TRIM21 response with as few as two antibodies per virion^11^. In order to understand what makes 9C12 a potent intracellular antibody and how it is able to stimulate activity at such low stoichiometry, we solved the 2.7 Å crystal structure of the 9C12 Fab in complex with full-length Adv5 hexon protein (Table 1). The crystal structure of the 9C12 Fab:hexon complex shows that binding takes place at the apex of the trimer spike, which is the most accessible part of hexon in the context of an assembled virion (Fig. 1a). This localization is consistent with previously published 20 Å cryo-EM data^12^. There is a single Fab and hexon per asymmetric unit, meaning that three Fabs can bind per trimer spike without steric clash. Adv5 hexon is comprised of several component domains, of which DE1 and FG1 are bound by 9C12 (Fig. 1b and c). Domains DE1 and FG1 are the least conserved parts of hexon and contain all adenoviral hyper-variable regions (HVRs); discrete linear sequences that have diversified extensively between serotypes^13^,^14^,^15^. Adv5 has nine HVRs in total, although the last three are sometimes regarded as a single HVR. These domains are also the most flexible; DE1 contains three dynamic regions that could not previously be modeled due to the absence of electron density in free hexon crystal structures^13^,^14^. In the 9C12 complexed structure however, two of these flexible regions have become fixed and can now be defined: residues 250–257, between strands β6a and β7 and residues 270–279, between strands β7 and β8 (Fig. 1c). This allows HVR4 and 5 respectively to be modeled for the first time. A third dynamic region in DE1, comprising the longest HVR (HVR1), has also become significantly more rigid in the 9C12 complex. In the free hexon structure there are 30 residues missing between 135–165; half of these residues are now resolved in the complex. The remaining missing piece, between 145–159, has an unusually negatively charged sequence (EEEDDDNEDEVDEQ) and forms the core of HVR1. The visible part of HVR1 forms a very extended loop that projects straight from the top of the spike. 9C12 makes no contact with this part of hexon and it is highly likely that any antibody targeting this region would pay a significant entropy cost and require highly positively charged complementarity-determining regions (CDRs). Domain FG1 also contains a flexible region (residues 430–437) that is missing in free hexon structures but has become fixed in the 9C12 complex. This sequence comprises HVR8 and is fully resolved in the 9C12 complexed structure.

**Figure 2:**
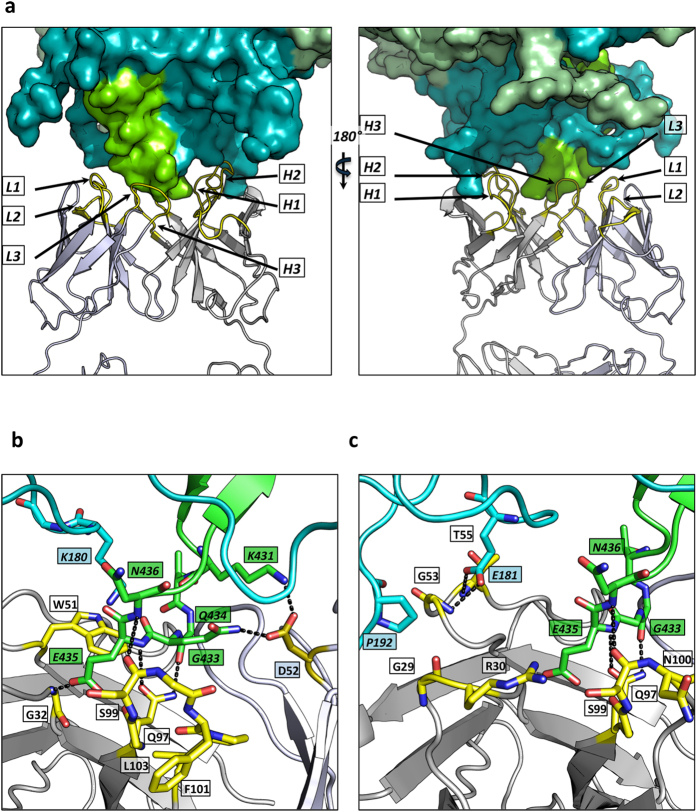
9C12 CDR interactions with hexon. (**a**) Surface representation of hexon (monomers in shades of green). Secondary structure of V_L_ (blue) and V_H_ (gray) with CDR loops marked in yellow. The 9C12 CDRs interact with HVRs from two hexon monomers. (**b**,**c**) Interacting residues from hexon (top, cyan & green) with residues from 9C12 (bottom, V_H_ in yellow, V_L_ in blue/gray). Dashed lines indicate putative hydrogen bonds. Two different views are shown corresponding to residues mutated in Figs 3(b) and 4(c).

The principal hexon epitopes bound by 9C12 are contained within HVRs 2 and 8 (Fig. 1d). Of these, HVR8 forms a finger-like projection into the center of the V_H_-V_L_ binding site, whilst HVR2 contacts V_H_ exclusively (Fig. 1c and d). With the exception of L2, all antibody CDR loops contribute to the interface (Fig. 2a). At one side of the interface, D52 from L2 makes a bifurcated hydrogen bond with residues K431 and Q434 from HVR8 (Fig. 2b). On the other side, E181 from HVR2 makes a bifurcated hydrogen bond with the peptidyl nitrogens of G53 and T55 from H2 (Fig. 2c). At the center of the interface, E435 of HVR8 hydrogen bonds with the main-chain of H1 residue G32 (Fig. 2b). A number of contacts are also made between HVR8 and H3, including between the side-chain of Q97 and the main chain of G433 and E435 and between the main-chain atoms of S99 and N436 (Fig. 2b). In addition to these hydrogen-bond interactions, W51 from H2 makes a cation-π interaction with K180 from HVR2 (Fig. 2b). Lysine-tryptophan cation-π interactions are less common than those involving arginine but stronger at 3.3 kcal/mol^16^.

**Figure 3:**
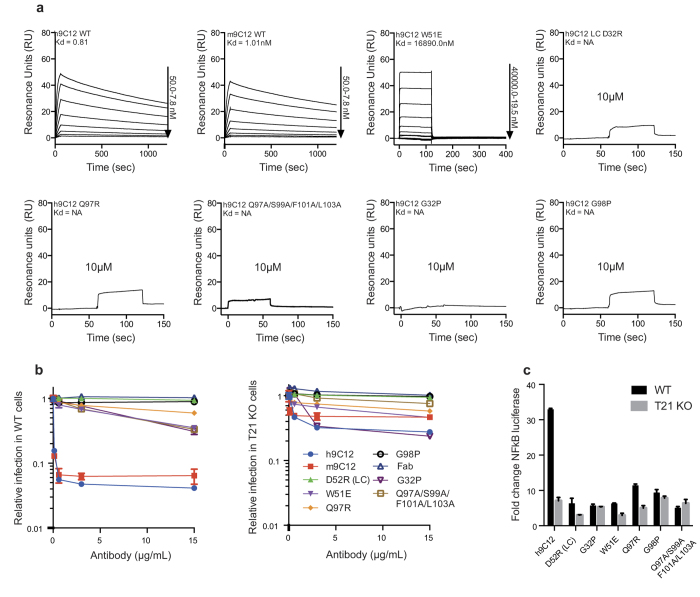
Structure-guided 9C12 mutants prevent hexon binding, Adv5 neutralization and NFκB activation. (**a**) SPR titrations to determine kinetics and affinity of mouse and human WT 9C12 and selected h9C12 CDR-engineered mutants that abrogate hexon binding. (**b**) Ability of h9C12 variants to prevent Adv5 infection in 293 T WT cells or 293 T TRIM21 KO cells. (**c**) NFκB induction by h9C12 variants in 293 T WT and TRIM21 KO cells upon adenovirus infection. Cellular experiments were performed in triplicate.

To determine the relative contribution of these interactions to 9C12:hexon binding, we introduced a number of mutations into the CDR loops of a recombinant humanized IgG1 version of 9C12 (h9C12) and measured relative binding using ELISA (Supplementary Figure 1). A range of activities was observed from close to wild-type (WT) to undetectable. Surface plasmon resonance (SPR) was used to determine 1:1 binding kinetics of Fab fragments from WT and CDR-engineered variants (Fig. 3a). In its original mouse format, 9C12 binds tightly to hexon with a Kd of 1 nM. We found that humanization made little difference to hexon binding (0.8 nM). This is consistent with previous data showing that h9C12 preserves its neutralization potency in human cell lines^17^. Mutations D52R in L2, G32P in H1 and Q97R or G98P in H3 abolished all measurable binding to hexon, confirming the importance of these residues as predicted by the crystal structure (Fig. 3a). Combined alanine mutations in H3 (Q97A/S99A/F101A/L103A) also prevented hexon binding (Fig. 3a).

Next, we tested how the affinity of h9C12 for hexon alters its ability to neutralize Adv5 and trigger NFκB activation during infection. The humanized antibody neutralized Adv5 infection ~25-fold in HEK293T cells, slightly more potently than the parental mouse antibody (Fig. 3b). Importantly, neutralization was significantly diminished in HEK293T cells where TRIM21 had been knocked-out (KO) using the CRISPR/Cas9 system^18^ (Fig. 3b). These results are consistent with published studies that demonstrate an important role for the cytosolic Fc receptor in viral neutralization^3^. Residual neutralization was greater for humanized versus mouse 9C12, suggesting that the greater potency of h9C12 is the result of a modest gain in TRIM21-independent neutralization activity. Importantly, all mutants that abolished measurable hexon binding displayed a dramatic reduction in neutralization activity (Fig. 3b). Remaining neutralization activity was observed for some mutants but, with the exception of Q97R, this was further diminished in TRIM21 KO cells. Consistent with the neutralization data, h9C12 hexon binding mutants also provoked significantly reduced TRIM21-dependent NFκB activation during infection (Fig. 3c). Taken together, this data suggests that in order for TRIM21 to mediate neutralization and immune activation during viral infection, antibody must be present in the form of a virus-antibody immune complex. Thus, TRIM21 antiviral activity fulfills the criteria for neutralization that it is a process strictly dependent upon binding of antibody to virus.

**Figure 4:**
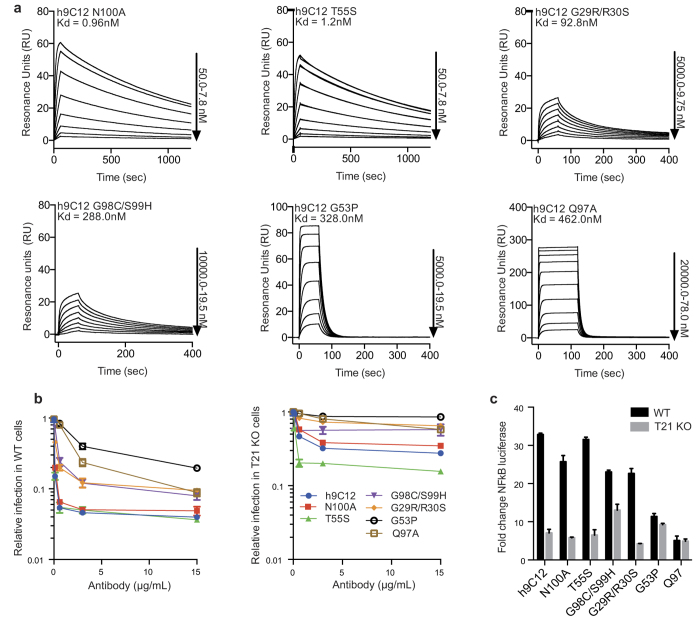
Modulation of h9C12 on and off-rate kinetics have disproportionate affects on antiviral activity. (**a**) SPR titrations of selected h9C12 variants. (**b**) Neutralization of Adv5 infection by h9C12 variants in 293 T WT cells or 293 T TRIM21 KO cells. (**c**) Induction of NFκB in 293 T WT and TRIM21 KO cells by h9C12 variants upon adenovirus infection. Cellular experiments were performed in triplicate.

In order to investigate how the kinetics of antigen binding influences TRIM21 activity we sought CDR mutations that modulated interaction without abolishing it. Binding of WT h9C12 to hexon is characterized by an on-rate constant of 6.35 × 10^5^ M^−1 ^s^−1 ^and an off-rate constant of 0.00052 s^−1^ (Fig. 3a). Mutations N100A and T55S had only marginal impact on binding kinetics and maintained ~1 nM Kd to hexon (Fig. 4a). Consistent with the binding data, these mutations had no significant impact on TRIM21-dependent neutralization or NFκB activation (Fig. 4b and c). Mutations G29R/R30S and G98C/S99H both reduced the off-rate constant by 10-fold and the on-rate constant ~10-fold and ~30-fold, respectively. Both mutants also showed an intermediate phenotype for TRIM21 dependent neutralization and NFκB activation (Fig. 4b and c). Q97A represented a threshold variant where only the affinity could be measured as the kinetics were too fast to determine (Fig. 4a and Supplementary Figure 1). With a Kd of 462 nM, Q97A had the weakest affinity of the antibody mutants tested; however, it did not have the least potent TRIM21 dependent neutralization. The mutant that was least protective in neutralization was G53P, despite the fact that it demonstrated higher affinity for hexon than Q97A and its on-rate constant was only 2.6 fold reduced compared to WT (Fig. 4a and b). However, G53P displayed a 152-fold increase in off-rate. This suggested that antigen binding kinetics may be an important determinant of TRIM21 activity. Further comparison of mutant activity and their kinetics provided additional evidence. G53P had significantly reduced activity in both neutralization and NFκB assays with respect to G98C/S99H, even though both mutants had similar affinity for hexon. The most striking difference between these two mutants is their variability in off-rate, which is much faster for G53P (Fig. 4a and Table 2). Conversely, G98C/S99H and G29R/R30S had very similar neutralization and NFκB activities, despite having very different affinities for hexon (Fig. 4b and c). Rather, the similar activities are predicted by their off-rates, which were almost identical (Fig. 4a). Thus, off-rate rather than on-rate is crucial for TRIM21 function. Indeed, if on-rate were the critical factor, then G53P would be one of the most active mutants rather than the weakest.

**Figure 5:**
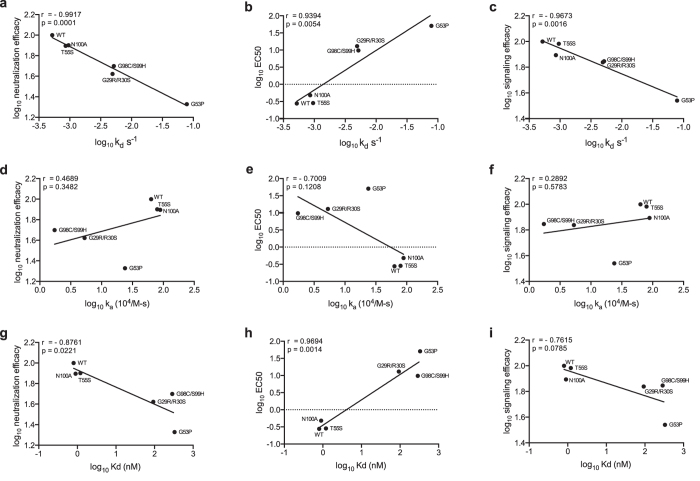
TRIM21-dependent neutralization and signaling is independent from on-rate but proportional to off-rate. Neutralization and signaling efficacy, expressed as percentage of WT activity, and the EC50 of selected h9C12 variants plotted against their off rates (**a**–**c**), on rates (**d**–**f**) and affinities (**g**–**i**) as determined by SPR. The Pearson correlation (Pearson’s correlation coefficient r) and the statistical significance (two-tailed P value) between these kinetic measures are shown.

To confirm that off-rate of the antibody-antigen complex rather than on-rate determines TRIM21 antiviral activity, we compared the TRIM21-dependent neutralization and signaling of all h9C12 mutants for which we had kinetic data (Fig. 5a–i). For neutralization we examined the correlation between mutant kinetics and both the potency (EC50) and efficacy (maximal neutralization) of the response. When using either measure of neutralization we observed a significant correlation with off-rate (p-values of 0.0054 and 0.0001 respectively) but not with on-rate (p-values of 0.3193 and 0.1208 respectively). Plotting the Kd (kd/ka) also gave a significant correlation with both neutralization measures, albeit the fit to efficacy is substantially poorer than to off-rate. Plotting against signaling responses gave a similar pattern as neutralization, namely that the principle correlate was off-rate, with correlation to affinity also statistically significant but on-rate non-significant. As our kinetic data were obtained using Fab fragments they represent intrinsic rate constants and do not report on any avidity contribution that may result from bivalent binding to virions. To assess whether avidity alters the relative potency of intermediate antibody mutants we compared binding of IgG and Fab fragments to intact adenovirus virions by ELISA. The IgGs had enhanced binding suggesting that bivalent binding is possible, consistent with the arrangement predicted by the crystal structure (Supplementary Figure 2). However, relative binding between mutants was broadly the same between IgG and Fab and a similar p-value was obtained for the correlation with neutralization efficacy.

## Discussion

Intensive research over many decades has determined what makes an antibody effective in classical extracellular humoral immunity. The characteristics of viral epitopes, in particular their accessibility and variability, are key factors. Equally important are the kinetics of antigen binding, which influence how potently antibodies stimulate immunity and prevent infection. Here we have defined the properties that determine how efficiently antibodies induce intracellular humoral immunity as mediated by the cytosolic antibody receptor TRIM21. Intracellular humoral immunity is distinct from other antibody mechanisms because it takes place in the cytosol, separated from the reservoir of serum antibodies. In order for an antibody to stimulate an immune response via TRIM21, it must bind to a pathogen extracellularly and then accompany the pathogen as it is captured by cell surface receptors, endocytosed and delivered into the cytosol. Our results show that this process places strict requirements on an antibody if it is to activate TRIM21.

Solving the crystal structure of the potently neutralizing and TRIM21-dependent anti-Adv5 9C12 Fab fragment in complex with Adv5 hexon protein revealed that binding takes place at the apex of the viral spike. Cryptic epitopes are known to disfavor antibody binding and may inhibit antibody receptor engagement by orientating the antibody molecule such that the Fc is poorly accessible. The ability of 9C12 to project unhindered from the adenoviral surface may explain why this antibody is so efficient at recruiting TRIM21 to incoming viral particles. Like many viral structural proteins, hexon is highly variable with nine HVRs that diverge in sequence between adenovirus serotypes. Perhaps unsurprisingly, these HVRs map to flexible loop regions and consequently it has not been possible to fully define their conformation in previous hexon structures. Using our 9C12:hexon structure we have been able to resolve the structure of HVRs 4, 5 and 8 and provide a partial conformation for HVR1. Importantly, it is the targeting of several of these HVRs, namely HVRs 2 and 8, which allows the Fab domains of 9C12 to bind hexon while the Fc remains accessible. Nevertheless, engagement of these structurally variable epitopes likely entails an entropy cost, which may explain why 9C12 does not display a particularly fast on-rate in its association with hexon. However, the relative insensitivity of TRIM21 activity to on-rate means that this does not impact on 9C12 potency. Indeed, independence from on-rate kinetics may be a crucial feature of intracellular humoral immunity and mitigate the problem of targeting dynamic epitopes. Entropy costs are paid in on-rate kinetics.

Using our 9C12:hexon structure, we have made a series of CDR-mutations with a range of affinities for hexon that differ by >20,000-fold (Kd from 0.8 nM to 17 μM), on-rate constants that vary by ~50-fold (1.8 to 88.9 × 10^4^ M^−1^ s^−1^) and off-rate constants that vary by >175-fold (4.5 × 10^−4^ to 7.9 × 10^−2^ nM). These mutants were introduced in a mouse-human chimeric version of 9C12 to define which properties determine how efficiently antibodies neutralize Adv5 infection and activate NFκB in a TRIM21-dependent manner. Our results clearly show that off-rate is by far the most critical parameter, with both neutralization and signaling efficiency dropping proportionally as the off-rate increases. Signaling is perhaps the most sensitive of these processes to altered off-rate kinetics, with fast off-rate mutant G53P displaying negligible TRIM21-dependent NFκB activation despite having a higher overall affinity than some active mutants. This is consistent with previously published data showing that signaling activity is more sensitive than neutralization to disruption of the TRIM21 binding site on IgG Fc^17^. It is not clear to what extent antibody can stimulate TRIM21 once dissociated from antigen. However, the fact that antibody-antigen off-rates correlate with the strength of immune activation suggests that free antibody is significantly less agonistic. The binding of TRIM21 itself to antibody occurs with high affinity and only a few antibodies are necessary to provoke a neutralization response^11^. TRIM21 has highest affinity for IgG but is also capable of binding IgM and IgA, albeit with reduced affinity (17 μM and 50 μM respectively to monomeric PRYSPRY domain)^1^,^2^,^3^,^19^. Given that full-length dimeric TRIM21 increases affinity to IgG by ~375-fold, the functional affinity to IgA and IgM is likely to be submicromolar. Indeed, both IgM and IgA can be used by TRIM21 to mediate both neutralization and signaling. It is not known whether TRIM21 can bind to and use IgE or IgD. TRIM21 uses a ring of hydrophobic residues to desolvate a short loop that is found in different antibody isotypes, which may explain its broad specificity.

The relative insensitivity of TRIM21 function to antibody:antigen on-rate may reflect the fact that activity is triggered primarily by the infection of pre-formed virus-antibody complexes. Re-association of dissociated complexes in the cytosol may be possible but is likely to be less efficient than in serum, where it can be promoted by excess free antibody. In contrast, it is intuitive that virus-antibody complexes with slow off-rates will be more likely to persist during the infection process to engage with TRIM21 once they reach the cytosol. Nevertheless, viruses posses different entry mechanisms and kinetics meaning that absolute values of threshold off-rates for TRIM21 activity may vary. However, given that adenovirus undergoes a relatively rapid cell entry process it is likely that off-rate will be at least as important if not more so for most other viruses.

Off-rate kinetics are known to be an important parameter in classical neutralization and have been shown to inversely correlate with neutralization titer after vaccination^20^. Given the importance of antibody-antigen off-rate, it is fortunate and perhaps unsurprising that B cell receptor activation has a threshold occupancy that selects against binders with rapid dissociation rates^21^. Furthermore, affinity maturation has been proposed^22^ and demonstrated^23^ to improve antigen off-rates independently of on-rate selection. The ability to improve antibodies kinetically rather than merely thermodynamically may be an important factor in the race against fast-evolving pathogens. The capacity to independently drive improvements in on- and off- rates may help counteract the viral strategy of masking important epitopes through intrinsic disorder^24^. However, improvements in off-rate have to contend with the limiting rate of B cell endocytosis^23^. A cross-antigenic process, whereby mutations concomitantly improve the kinetics to related antigens, has been proposed to explain why very slow off-rate neutralizing antibodies routinely evolve^25^. Intracellular applications for therapeutic antibodies are as yet in their infancy but our results help define the properties that are required to drive efficient cytosolic immunity via TRIM21. These lessons from nature teach that a strategy of off-rate screening^26^ would be the most effective approach to generate potent TRIM21-dependent antibodies.

## Methods

### Recombinant h9C12 and Fab fragments

Vectors encoding h9C12 IgG1 variants were constructed by synthesizing gene fragments containing the desired mutations (Genscript, USA) and sub-cloned into either ^h9C12^pLNOH2-hIgG1-WT-OriP or ^h9C12^pLNOκ-hLC using the restriction sites BsmI and BsiWI (Genscript, USA) as previously described^27^. Gene fragments encoding 9C12 and h9C12 Fab fragments were synthesized as cDNA by truncating the h9C12 HC after the codon corresponding to the first hinge cysteine (C220; EU numbering) and introduction of a stop codon. The cDNA fragments were then sub-cloned into ^h9C12^pLNOH2-hIgG1-WT-OriP using the restriction sites BsmI and BamHI, replacing the full-length h9C12 sequence. Vectors encoding h9C12 IgG1 and h9C12 Fab variants were transiently co-transfected together with the LC vector into HEK293E cells using Lipofectamine2000 (ThermoFisher, USA). The proteins were purified using a CaptureSelect C_H_1 specific column (ThermoFisher, USA), and monomeric fractions were isolated by size exclusion chromatography using a Superdex 200 10/300 or a Superdex 75 10/300 column for full-length h9C12 and h9C12 Fab fragments, respectively. Purified proteins were analyzed by SDS-PAGE (ThermoFisher, USA).

### Enzyme-linked immunosorbent assay (ELISA)

96-well EIA/RIA plates (Corning Costar, USA) were coated with either 100 μl recombinant AdV5 hexon protein (AbD Serotech, USA) or a polyclonal anti-human Fc (hFc) Ab from goat (locally produced/1 μg/ml in PBS) and incubated over night at 4 °C. The plates were blocked for 2 hours with 250 μl PBS containing 4% skimmed milk powder (S) (Sigma Aldrich, USA) dissolved in PBS (S/PBS), followed by washing four times with PBS containing 0.05% Tween20 (T) (Sigma Aldrich, USA). Next, titrated amounts of purified h9C12 variants diluted in S/PBS/T were added to the plates and incubated for 1 hour on a shaker. After washing as above, 100 μl alkaline phosphatase (ALP)-conjugated anti-hFc Ab from goat (Sigma Aldrich, USA) (1 μg/ml in PBS/S/T) were added and incubated for 1 hour at room temperature. Following washing, bound proteins were visualized by adding 100 μl ALP substrate (1 mg/ml) dissolved in diethanolamine buffer (Sigma Aldrich, USA). The 405 nm absorbance spectrum was measured using a Sunrise spectrophotometer (Tecan, Switzerland).

### SPR

A Biacore T200 instrument (GE Healthcare, USA) was used for all kinetics measurements. Recombinant AdV5 hexon protein (AbD Serotech) was immobilized by amine coupling on a CM5 sensor chip according to the manufacturer’s instructions. Covalent immobilization may alter affinity for hexon and an assumption of our analysis is that any conformational disturbance is proportional among the mutant Fabs being measured. The coupling was performed by injecting 5–10 μg/ml hexon dissolved in 10 mM sodium acetate (pH 4.5). Unreacted surface moieties were blocked by injecting 1 M ethanolamine. Kinetics measurements were performed by injecting titrated amounts of monomeric h9C12 Fab fragments over the immobilized hexon. HBS-P (0.01 M HEPES, 0.15 M NaCl, 0.005% surfactant P20, pH 7.4) was used as both running and dilution buffers. Affinity constants were determined using the Biacore T200 evaluation software (GE Healthcare, USA) by fitting the binding responses to a simple first order (1:1) Langmuir bio-molecular interaction model. For low affinity interactions, steady state constants were obtained using an equilibrium (R_eq_) binding model.

To minimize the influence of mass transport or re-binding, the lowest level of the hexon protein that made it possible to directly compare binding of all IgG Fab variants using the same chip was immobilized. The flow rate was set to 50 μl/min, except for the three variants that bound with fast kinetics where the flow rate was 30 μl/min and the binding data fitted to the steady state affinity model.

### Generation of knockout cell lines using the CRISPR/Cas9 system

HEK293T cells were seeded in 6-well plates (2.5 × 10^5^ cell/well) and allowed to adhere over night. Cells were transfected with 1 μg pX458 (Addgene, USA)^18^ encoding the desired gRNA. 24 hours post transfection cells were trypsinized and GFP positive cells were sorted into 96-well plates (1 cell/well) using FACS. 14 days after sorting HEK293T clones were assessed for an indel event by Western Blot and Sanger Sequencing.

### Cell culture

HEK293T cells were maintained in DMEM supplemented with 10% FBS, 100 U/ml penicillin, and 100 mg/ml streptomycin at 37 °C in a humid 5% CO2, 95% air incubator.

### Neutralization assay

HEK293T cells were plated at a density of 5 × 10^4 ^cells/well in 24-well plates and were allowed to adhere over night. 6 × 10^7^ infectious units AdV5-mCherry/mL (ViraQuest, USA) were mixed 1:1 with antibody (at the indicated concentration), and incubated for 30 min at room temperature to allow for complex formation. 12 μL of the virus-antibody complex was added per well and allowed to incubate for 24 hours at 37 °C. Cells were then collected by trypsinization, acquired on a BD LSRII flow cytometer (BD Biosciences, USA) and analyzed for mCherry gene expression using FlowJo software (FlowJo LLC, USA). Relative infection was calculated as described^11^.

### NFκB reporter assay

HEK293T cells were transiently transfected with pGL4.32 NFκB luciferase (Promega, USA) using Fugene 6 (Promega, USA). 24 hours post transfection cells were plated at a density of 1 × 10^4 ^cells/well in clear-bottom 96-well plates (Corning, USA) and were allowed to adhere over night. 3 × 10^10^ infectious units AdV5-mCherry/mL (ViraQuest, USA) were mixed 1:1 with antibody (20 μg/mL), and incubated for 30 min at room temperature to allow for complex formation. 5 μL of the virus-antibody complex was added to each well and allowed to incubate for 7 hours at 37 °C before the addition of 100 μL steadylite plus luciferase reagent (Perkin Elmer, USA) and analysis using a BMG PHERAstar FS plate reader.

### Crystallization

9C12 was purified from hybridoma cells using Protein A and Fab fragments produced by papain proteolysis. Hexon was purified from Adv5-infected cells by CsCl-gradient and size exclusion. 9C12 and hexon were mixed 1:1 to obtain a 5 mg/ml complex. Crystals were grown by vapour diffusion in 15% MPD, 5% PEG 4 K, 0.1 M imidazole pH 8 and frozen at 100 K. Data was collected at the DIAMOND synchrotron on beamline I03, integrated using MOSFLM and indexed using SCALA^28^. Model 3TG7 was used to obtain a molecular replacement solution in PHASER^29^ and subsequent refinement was carried out in Coot^30^ and REFMAC^28^. The final refined structure and data were deposited under accession code 5LDV.

### Statistical analysis

Figures were generated and statistical analyses were performed using GraphPad Prism version 6.0 (GraphPad, USA). All h9C12 variants were tested at least three times, and error bars represent SD from one representative experiment. The neutralization and signaling efficacy data points used in Pearson correlation analysis represent the mean of triplicate measurements within one experiment. EC50 values were determined using a nonlinear regression curve fit constraining the maximum activity to 1.

## Additional Information

**How to cite this article**: Bottermann, M. *et al*. Antibody-antigen kinetics constrain intracellular humoral immunity. *Sci. Rep.* **6**, 37457; doi: 10.1038/srep37457 (2016).

**Publisher's note:** Springer Nature remains neutral with regard to jurisdictional claims in published maps and institutional affiliations.

## Supplementary Material

Supplementary Information

## Figures and Tables

**Table 1:** Data collection and refinement statistics.

	5LDN
**Data collection**	
Space group	P63
Cell dimensions	
*a, b, c* (Å)	157.0, 157.0, 144.9
α, β, γ (°)	90.0, 90.0, 120.0
Resolution (Å)	78.5–2.7 (2.77–2.7)
*R*_meas_	0.167 (0.964)
*I*/σ*I*	8.6 (1.1)
Completeness (%)	99.5 (98.9)
Redundancy	4.6 (4.4)
**Refinement**	
Resolution (Å)	2.7
No. reflections	55619
*R*_work_/*R*_free_	0.16/0.22
No. atoms	11253
Protein	
Ligand/ion	10773
Water	480
*B*-factors	
Protein	41.1
Ligand/ion	—
Water	44.2
R.m.s. deviations	
Bond lengths (Å)	0.009
Bond angles (°)	0.84

^*^Values in parentheses are for highest-resolution shell.

**Table 2:** SPR-derived binding kinetics for h9C12 Fab variants to Adv5 hexon.

Variant	k_a_ (10^4^/Ms)^a^	k_d_ (1/s)^a^	Kd (nM) (1:1)^a^	Steady state affinity^b^
9C12	44.7	4.5E^−4^	1.0	ND^d^
h9C12	63.5	5.2E^−4^	0.8	ND
N100A	88.9	8.5E^−4^	0.9	ND
T55S	79.5	9.5E^−4^	1.2	ND
G29R/R30S	5.3	49.0E^−4^	92.8	ND
G98C/S99H	1.8	52.0E^−4^	288.0	ND
G53P	23.9	7.9E^−2^	330.0	ND
Q97A	NA^c^	NA	NA	462.0
W51E	NA	NA	NA	16890.0
G98P	NA	NA	NA	NA
Q97R	NA	NA	NA	NA
G32P	NA	NA	NA	NA
WT + LC D52R	NA	NA	NA	NA
Q97A/S99A/F101A/L103A	NA	NA	NA	NA

^a^The kinetic rate constants were obtained using a simple first-order (1:1) Langmuir bimolecular interaction model. The kinetic values represent the average of triplicates.

^b^The steady-state affinity constants were obtained using an equilibrium (Req) binding model supplied by the BIAevaluation 4.1 software. The affinities derived from equilibrium binding data represent the average of triplicates.

^c^NA, not acquired because of fast binding kinetics.

^d^ND, not determined.
