# Corrigendum: Antibody-antigen kinetics constrain intracellular humoral immunity

**DOI:** 10.1038/srep45418

**Published:** 2017-04-06

**Authors:** Maria Bottermann, Heidrun Elisabeth Lode, Ruth E. Watkinson, Stian Foss, Inger Sandlie, Jan Terje Andersen, Leo C. James

Scientific Reports 6: Article number: 3745710.1038/srep37457; published online: 11 24 2016; updated: 04 06 2017

This Article contains a typographical error in the Methods section under the subheading ‘Crystallization’, where the Protein Data Bank accession code ‘5LDN’ was incorrectly given as ‘5LDV’.

