# Supplementary material for: Antibody-antigen kinetics constrain intracellular humoral immunity
Source: Sci Rep. 2016 Nov 24;6:37457. doi: 10.1038/srep37457 (PMC5121590; doi:10.1038/srep37457)
Supplement: Supplementary Information [file srep37457-s1.pdf]

Antibody-antigen kinetics constrain intracellular humoral immunity

Maria Bottermann[1§], Heidrun Elisabeth Lode[2§], Ruth E. Watkinson, Stian Foss[2], Inger Sandlie[2], Jan Terje Andersen[2*], Leo C. James[1*]

[1]Medical Research Council Laboratory of Molecular Biology, Division of Protein and Nucleic Acid Chemistry, Francis Crick Avenue, Cambridge, CB2 0QH, United Kingdom.

2[1]Centre for Immune Regulation, Department of Biosciences, University of Oslo, N-0371 Oslo, Norway; Centre for Immune Regulation, Department of Immunology, Oslo University Hospital Rikshospitalet, University of Oslo, N-0424 Oslo, Norway

[§]These authors contributed equally

*To whom correspondence should be addressed: lcj@mrc-lmb.cam.ac.uk & j.t.andersen@medisin.uio.no

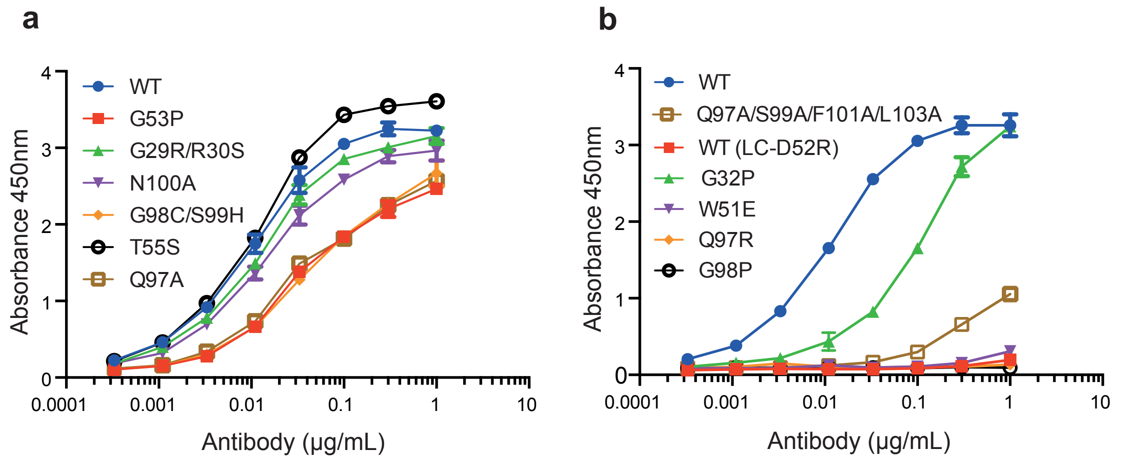

Supplementary Figure 1: Affinity determined by ELISA. Antibody titrations of CDR mutants that abrogate **(a)** and modulate **(b)** hexon binding, determined by ELISA.

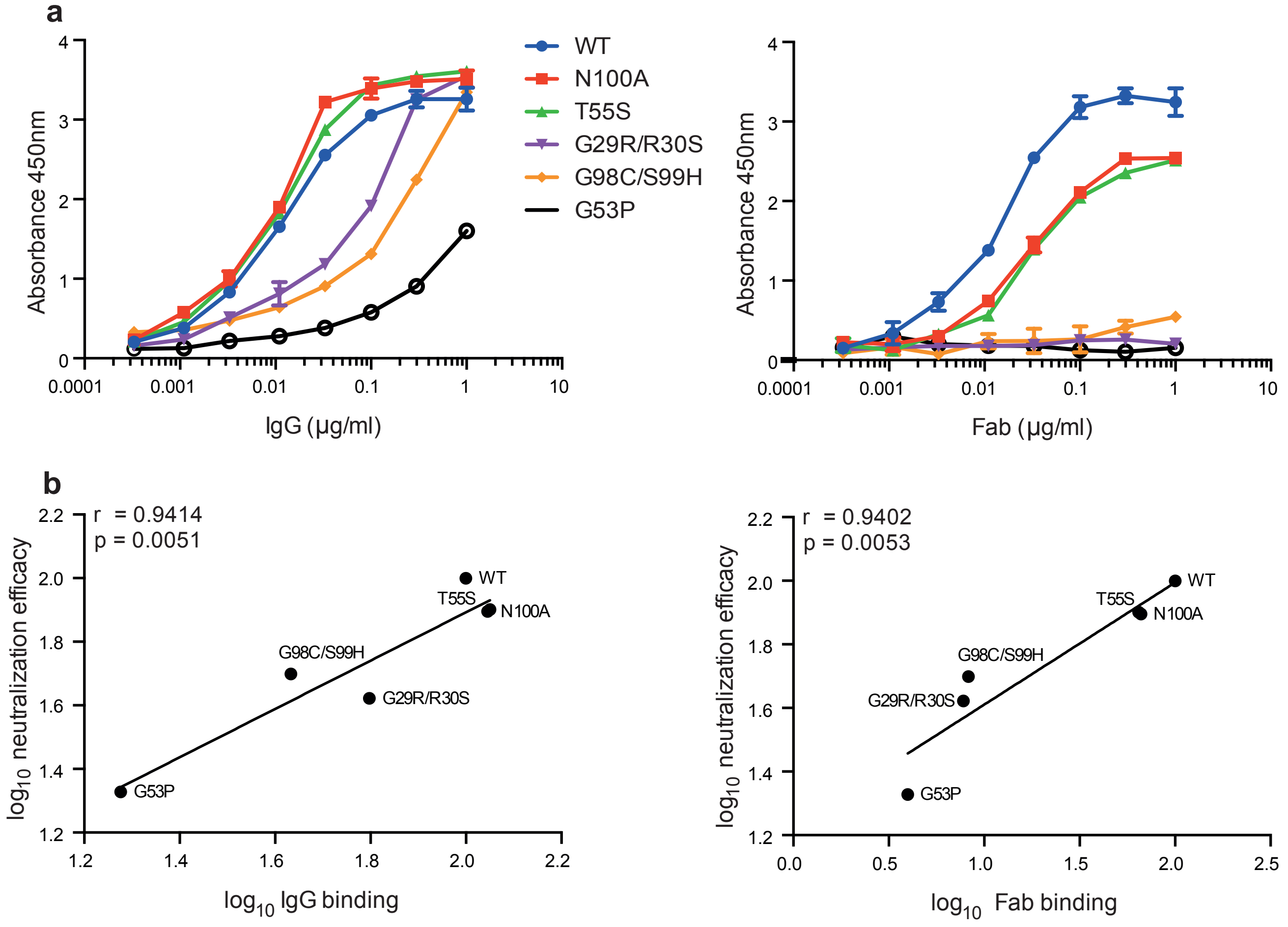

Supplementary Figure 2: Avidity increases antibody binding to Adv5 proportionally to intrinsic affinity. (a) Comparison of IgG and Fab fragment binding to Adv5 determined by Elisa. **(b)** IgG and Fab fragment binding expressed as percentage of WT binding plotted against neutralization efficacy. The Pearson correlation (Pearson's correlation coefficient r) and the statistical significance (two-tailed p-value) between these kinetic measures are shown.
